# Fecal Microbiota Composition, Their Interactions, and Metagenome Function in US Adults with Type 2 Diabetes According to Enterotypes

**DOI:** 10.3390/ijms24119533

**Published:** 2023-05-31

**Authors:** Sunmin Park, Ting Zhang, Suna Kang

**Affiliations:** 1 Department of Food and Nutrition, Obesity/Diabetes Research Center, Hoseo University, 165 Sechul-Ri, Asan 31499, Republic of Korea; roypower003@naver.com; 2 Department of Bioconvergence, Hoseo University, Asan 31499, Republic of Korea; zhangting92925@gmail.com

**Keywords:** fecal bacteria, type 2 diabetes, pooling data, western diet, metagenome analysis

## Abstract

T2DM etiology differs among Asians and Caucasians and may be associated with gut microbiota influenced by different diet patterns. However, the association between fecal bacterial composition, enterotypes, and T2DM susceptibility remained controversial. We investigated the fecal bacterial composition, co-abundance network, and metagenome function in US adults with T2DM compared to healthy adults based on enterotypes. We analyzed 1911 fecal bacterial files of 1039 T2DM and 872 healthy US adults from the Human Microbiome Projects. Operational taxonomic units were obtained after filtering and cleaning the files using Qiime2 tools. Machine learning and network analysis identified primary bacteria and their interactions influencing T2DM incidence, clustered into enterotypes, Bacteroidaceae (ET-B), Lachnospiraceae (ET-L), and Prevotellaceae (ET-P). ET-B showed higher T2DM incidence. Alpha-diversity was significantly lower in T2DM in ET-L and ET-P (*p* < 0.0001), but not in ET-B. Beta-diversity revealed a distinct separation between T2DM and healthy groups across all enterotypes (*p* < 0.0001). The XGBoost model exhibited high accuracy and sensitivity. *Enterocloster bolteae*, *Facalicatena fissicatena*, *Clostridium symbiosum*, and *Facalibacterium prausnitizii* were more abundant in the T2DM group than in the healthy group. *Bacteroides koreensis*, *Oscillibacter ruminantium*, *Bacteroides uniformis*, and *Blautia wexlerae* were lower in the T2DM than in the healthy group regardless of the enterotypes in the XGBoost model (*p* < 0.0001). However, the patterns of microbial interactions varied among different enterotypes affecting T2DM risk. The interaction between fecal bacteria was more tightly regulated in the ET-L than in the ET-B and ET-P groups (*p* < 0.001). Metagenomic analysis revealed an inverse association between bacteria abundance in T2DM, energy utility, butanoate and propanoate metabolism, and the insulin signaling pathway (*p* < 0.0001). In conclusion, fecal bacteria play a role in T2DM pathogenesis, particularly within different enterotypes, providing valuable insights into the link between gut microbiota and T2DM in the US population.

## 1. Introduction 

Type 2 diabetes (T2DM) is a metabolic disease characterized by elevated serum glucose concentrations due to insulin resistance and impaired insulin secretion. The prevalence of T2DM has markedly increased among Asians [1] and is related to different etiology of T2DM among Asians and Caucasians [2]. In Asians, T2DM occurs in lean individuals with lower insulin-secreting capacity and pancreatic β-cell mass, which eventually causes hyperglycemia and T2DM [3]. It occurs without hyperinsulinemia in Asians [3]. However, in Western countries, increased insulin resistance due to obesity, aging, and inflammation is overcome by hyperinsulinemia, which delays the progression to T2DM. Over time, the increased insulin secretion in a person with high insulin resistance causes β-cell exhaustion, leading to T2DM. Therefore, T2DM is progressed slowly through an increase in insulin secretion in adults in Western countries, unlike Asians [4]. 

The difference in T2DM etiology between Asians and Western individuals is linked to lifestyle factors across generations. Traditionally, Asians have consumed a high-carbohydrate diet rich in dietary fiber, resulting in greater insulin sensitivity but lower insulin-secreting capacity and pancreatic β-cell mass compared to Caucasians [5]. On the contrary, Western individuals living in the USA have traditionally adopted a diet high in meat consumption, leading to a higher prevalence of obesity and elevated fasting serum glucose and insulin concentrations [6,7]. These differences in dietary patterns and metabolic characteristics influence the composition of the gut microbiota, contributing to the etiology of T2DM [8,9]. Butyrate and propionate among SCFA, products of fecal bacteria, are beneficial for insulin sensitivity, and T2DM patients exhibit reduced production, potentially contributing to impaired glucose homeostasis [10]. *Akkermansia* is positive, but certain species of *Escherichia* and *Clostridium* are inversely associated with metabolic function, contributing to T2DM risk [11,12]. Therefore, they have been shown to exert beneficial effects on glucose metabolism and insulin sensitivity.

In recent years, the crucial role of gut microbiota in the disease pathophysiology of T2DM has emerged [8,9]. The metabolites and bacterial components of gut microbiota affect the initiation and progression of T2DM [13]. Genetic differences, lifestyles, and interactions influence gut microbiota linked to T2DM [14]. The emerging evidence on the interconnectedness between the gut microbiome and host metabolism indicates that gut microbiota is associated with intestinal permeability, secretion of digestive juices, and the autonomous nervous system, all linked to the host’s genetic predisposition [15]. In a hyperglycemic state, the acute stimulation of the vagus nerve, a principal component of the parasympathetic nervous system, decreases glucose release from the liver and potentiates insulin secretion from the pancreatic β-cells [16]. The vagus nerve activation suppresses peripheral inflammation, decreases intestinal permeability, and modulates the microbiota composition [11]. The fecal bacteria community of Asians with T2DM exhibits the potential association with vagus nerve suppression, suggesting that insufficient insulin secretion in Asian T2DM may be related to the inhibition of the vagus nerve. However, T2DM, mainly with insulin resistance in Western countries, may have different gut microbiota communities. 

Limited evidence exists regarding the link between fecal bacteria composition and T2DM, specifically in Western countries. Furthermore, the composition of fecal bacteria and metagenome function in adults with T2DM in the USA, which predominantly represents a Caucasian population, may exhibit differences compared to populations in Asia due to distinct T2DM etiologies between Asians and non-Asians [3]. In the present study, we aimed to identify and analyze the specific variations in gut microbiota associated with T2DM in the USA, categorized according to enterotypes by pooling the fecal bacteria data since the sample size of previous studies was too small to reveal consistent results in the association between fecal bacteria and T2DM in the USA. The present study can provide insight into T2DM etiology in the aspect of fecal bacteria in the USA. 

## 2. Results

### 2.1. Collection of Fecal Bacteria and Their Enterotypes

**Figure 1:**
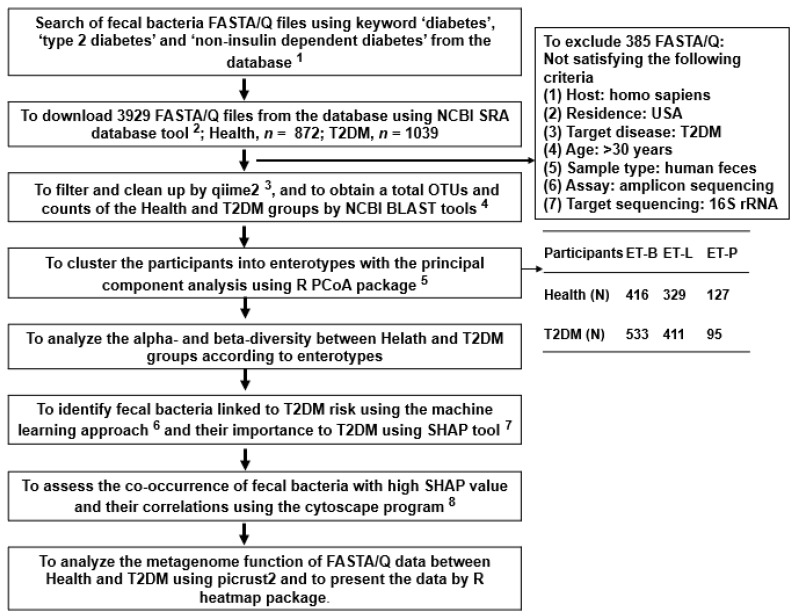
The scheme of the overall fecal FASTA/Q selection process and analysis methods. ^1^ SRA accession list on NCBI SRA database (https://www.ncbi.nlm.nih.gov/sra) (accessed on 6 and 10 March 2022) and Human Microbiome Project (https://portal.hmpdacc.org/) (accessed on 6 March 2022). ^2^ https://trace.ncbi.nlm.nih.gov/Traces/sra/sra.cgi?view=software (accessed on 10 March 2022). Bacteroidaceae (ET-B), Lachnospiraceae (ET-L), and Prevotellaceae (ET-P). ^3^ https://view.qiime2.org/ (accessed on 20 April 2022). ^4^ https://blast.ncbi.nlm.nih.gov/Blast.cgi (accessed on 3 May 2022). ^5^ https://cran.r-project.org/web/packages/aPCoA/index.html (accessed on 18 May 2022). ^6^ https://xgboost.readthedocs.io/en/stable/install.html (accessed on 25 May 2022). ^7^ https://shap.readthedocs.io/en/latest/index.html (accessed on 8 June 2022). ^8^ https://cytoscape.org (accessed on 20 June 2022).

A total of 1911 FASTA/Q fecal bacteria were collected: 872 and 1039 FASTA/Q belonged to the healthy and T2DM groups, respectively (Figure 1). The T2DM group included 611 men and 428 women, and the healthy group included 485 men and 387 women. Their average age was 44.6 ± 2.32 years for T2DM and 43.9 ± 1.53 years for the healthy group. They were clustered into three enterotypes determined with an eigenvalue > 1.5 using PCA analysis (Supplementary Figure S1A). The optimal number of clusters was three, represented by enterotypes, and three enterotypes were assigned and named based on the predominant bacterial family. A total of three clusters were named as enterotypes, namely: Bacteroidaceae (ET-B), Lachnospiraceae (ET-L), and Prevotellaceae (ET-P) (Supplementary Figure S1B). The ET-B included 416 healthy and 533 T2DM subjects, ET-L included 329 healthy and 411 T2DM subjects, and ET-P included 127 healthy and 95 T2DM subjects (Figure 1). 

The fecal bacterial composition of ET-B was 53.1% *Bacteroidaceae*, 10.6% *Lachnospiraceae*, 11.0% *Oscillospiraceae*, and 1.18% *Prevotellaceae*; that of ET-L was 22.1% *Oscillospiraceae*, 20.3% *Lachnospiraceae*, 19.5% *Bacteroidaceae*, and 1.72% *Prevotellaceae;* and ET-P was 43.4% *Prevotellaceae*, 14.5% *Bacteroidaceae*, 10.8% *Oscillospiraceae*, and 10.0% *Lachnospiraceae* (Supplementary Figure S2A). The ET-B predominantly included bacteria from the genera *Phocaeicola* and *Bacteroides* (*p* < 0.0001); ET-P was rich in *Prevotella* (*p* < 0.0001); ET-M included equal proportions of genera *Phocaeicola*, *Bacteroides*, and *Faecalibacterium* (*p* < 0.0001; Supplementary Figure S2B). 

### 2.2. Differences in the Bacterial Composition between the T2DM and Healthy Groups in Total Participants

**Figure 2:**
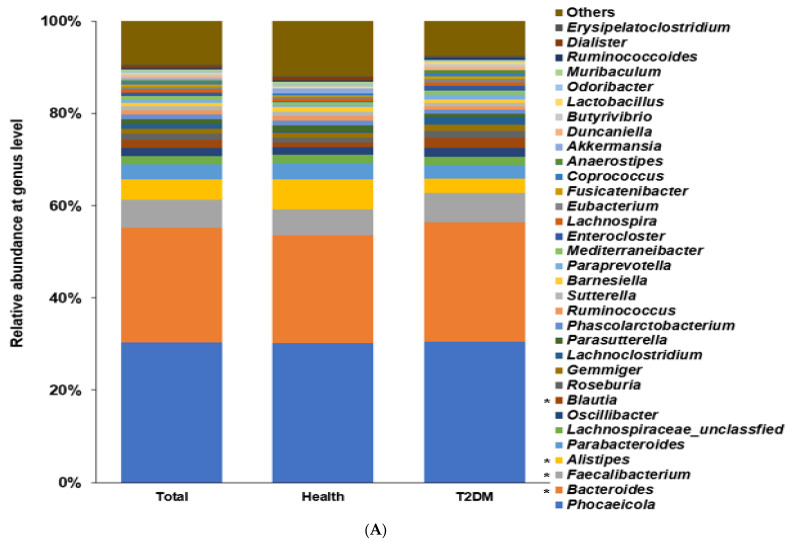

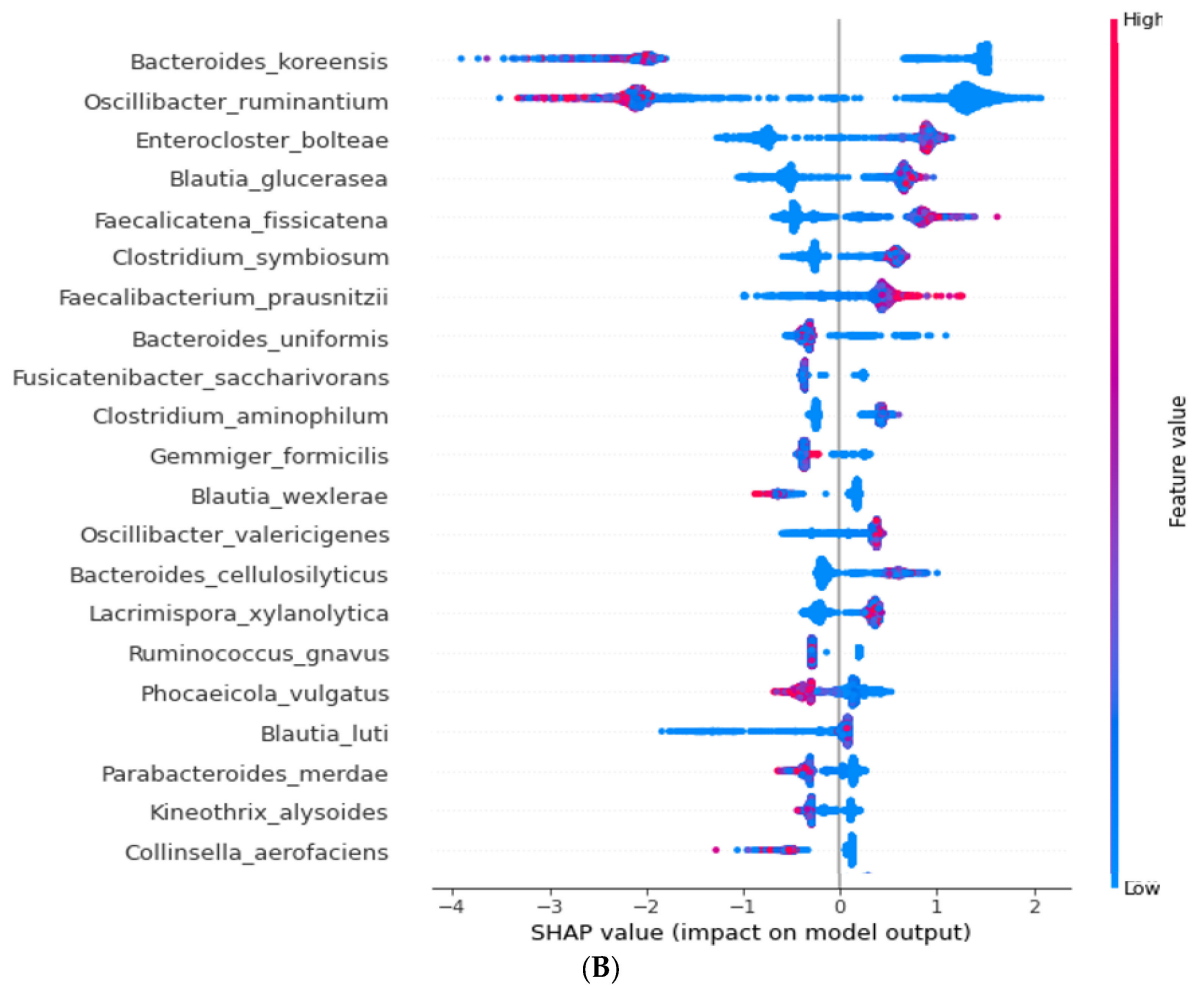
Fecal bacteria composition in total participants. Distinct differences in the abundance of specific bacterial genera between individuals with T2DM and the healthy group of US adults shed light on the potential role of these genera in T2DM pathogenesis. (**A**). Relative abundance of fecal bacteria compositions at the genus level between type 2 diabetes (T2DM) and healthy groups. (**B**). Primary bacteria between the type 2 diabetes (T2DM) and healthy groups at the species level by XGboost in each enterotype. * Significant differences between T2DM and healthy groups at *p* < 0.00001 (Bonferroni corrected *p* value).

Among the bacterial families present, the participants in the T2DM group showed a higher proportion of *Bacteroidaceae* and *Lachanospiraceae* than those in the healthy group (*p* < 0.0001; Supplementary Figure S3A). Among the genera, a higher abundance of *Bacteroides* and *Facalibacterium* and a lower proportion of *Allistipes* were present (*p* < 0.0001; Figure 2A). The α-diversity represented with the Chao1 (*p* < 0.0001), Shannon (*p* < 0.0001), and Simpson (*p* = 0.033) indexes was lower in the T2DM group than in the healthy group (Supplementary Figure S3A). The bacterial composition of the healthy group was well-separated and distinct from that of the T2DM group with a *p* < 0.001 (Supplementary Figure S4A). 

According to the machine learning approach, an optimal model to demarcate bacteria into the T2DM and healthy groups was explored. The area under ROC (AUROC) in the models estimated by XGboost, random forest, and linear regression were 1.0, 1.0, and 0.988, respectively, in total participants (Table 1). *Enterocloster bolteae*, *Facalicatena fissicatena*, *Clostridium symbiosum*, *Facalibacterium prausnitizii*, and *Oscillibacter valericigenes* were present in higher proportions in the T2DM group compared to the healthy group (*p* < 0.001), and *Bacteroides koreensis*, *Oscillibacter ruminantium*, *Bacteroides uniformis*, *Blautia wexlerae*, *Phocaeicola vulgatus*, and *Collinsella aerofaciens* were present in lower proportions in the T2DM group (*p* < 0.001; Figure 2B). 

### 2.3. Differences in the Bacterial Composition between the T2DM and Healthy Groups in ET-B

**Figure 3:**
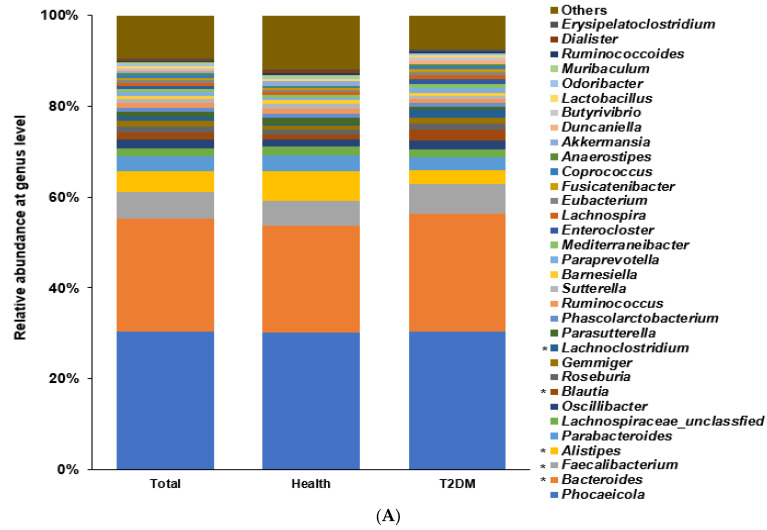

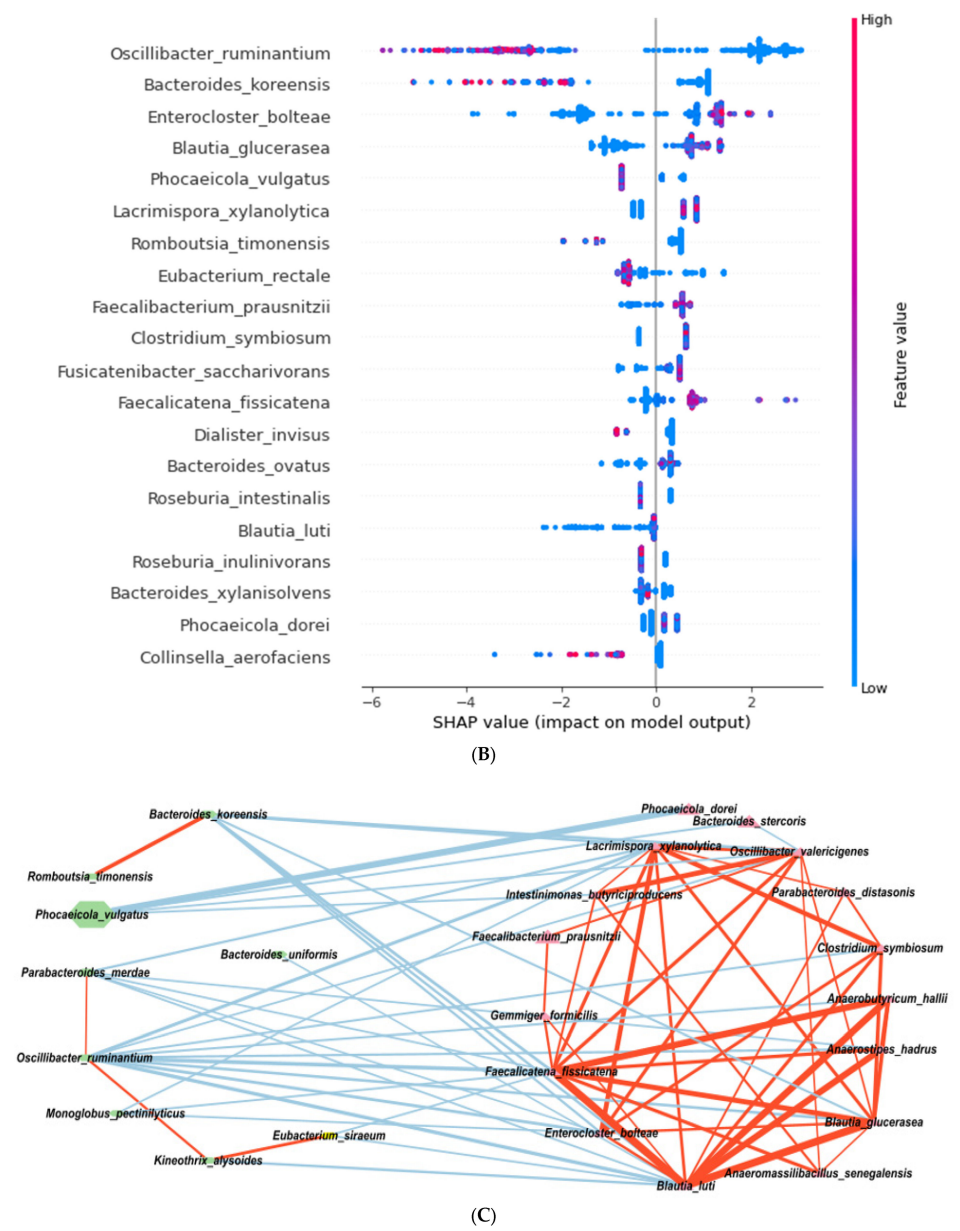
Fecal bacteria composition in Enterotype Bacteroidaceae (ET-B). In US adults with ET-B, the T2DM group exhibited a higher proportion of *Bacteroides*, *Facalibacterium*, *Lachnoclostridium*, and *Blautia*, while the proportion of *Allistipes* was lower in abundance than the healthy group. The interactions among bacterial families and genera were more robust in the T2DM group than in the healthy group. (**A**). Relative abundance of fecal bacteria compositions at the genus level between type 2 diabetes (T2DM) and healthy groups. (**B**). Primary bacteria between the type 2 diabetes (T2DM) and healthy groups at the species level by XGboost in each enterotype. (**C**). Network of primary bacteria in the T2DM and healthy groups according to each enterotype. Red and blue lines indicate positive and negative correlations with Pearson correlation coefficient > 0.1, respectively (*p* < 0.001). * Significant differences between T2DM and healthy groups at *p* < 0.00001 (Bonferroni corrected *p* value).

Within the ET-B, the T2DM group had a higher proportion of the family *Bacteroidaceae* and *Lachnospiraceae* and a lower proportion of *Rikenellaceae* than the healthy group. The T2DM group had a higher proportion of genera *Bacteroides*, *Facalibacterium*, *Lachnoclostridium*, and *Blautia* and lower *Allistipes* than the healthy group (*p* < 0.0001; Figure 3A). The Ace, Chao1, and Shannon indexes representing α-diversity were not different between the healthy and T2DM groups in the adults with ET-B (*p* > 0.05; Supplementary Figure S3B). However, β-diversity results revealed that the bacterial community was well-demarcated into the healthy and T2DM groups at *p* < 0.001 (Supplementary Figure S4B). 

According to the machine learning approach, an optimal model to separate bacteria between the T2DM and healthy groups was explored. The AUROC in the models estimated by XGboost, random forest, and linear regression were 0.998, 0.997, and 0.994, respectively (Table 1)*. Enterocloster bolteae*, *Facalicatena fissicatena*, *Clostridium symbiosum*, *Facalibacterium prausnitizii*, *Oscillibacter valericigenes*, *Clostridium symbiosum*, and *Bacteroides cellulosilyticus* were present in higher proportions in the T2DM than the healthy group (*p* < 0.001), and *Bacteroides koreensis*, *Oscillibacter ruminantium*, *Bacteroides uniformis*, *Blautia wexlerae*, *Phocaeicola vulgatus*, and *Eubacterium rectale* were present in lower proportions in the T2DM compared to the healthy group (*p* < 0.0001; Figure 3B). 

In the healthy group, certain bacteria showed positive correlations with other bacteria within the same group, but negative correlations with those in the T2DM group (*p* < 0.001; Figure 3C). The bacterial families and genera in the T2DM group exhibited stronger interactions compared to the healthy group, implying that there was a higher level of interconnectedness or interdependence among the bacteria in the T2DM group. 

### 2.4. Differences in the Bacterial Composition between the T2DM and Healthy Groups in ET-L

**Figure 4:**
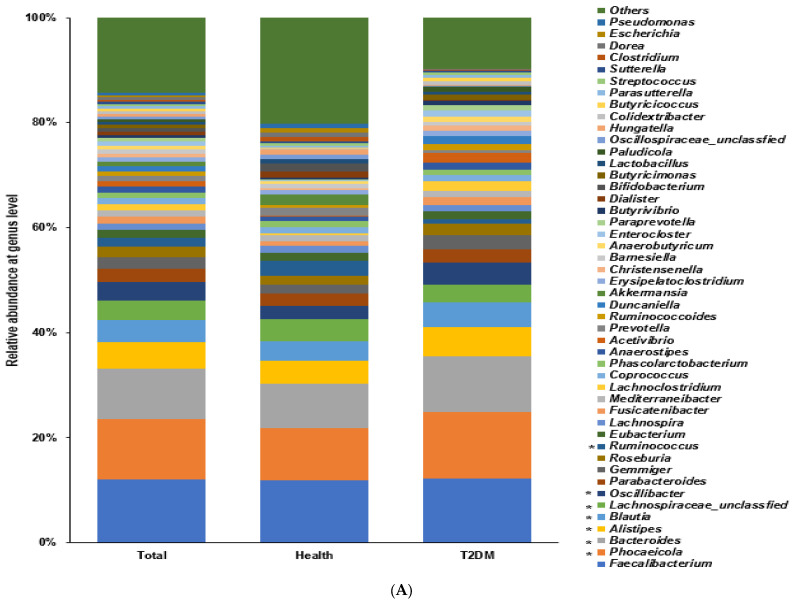

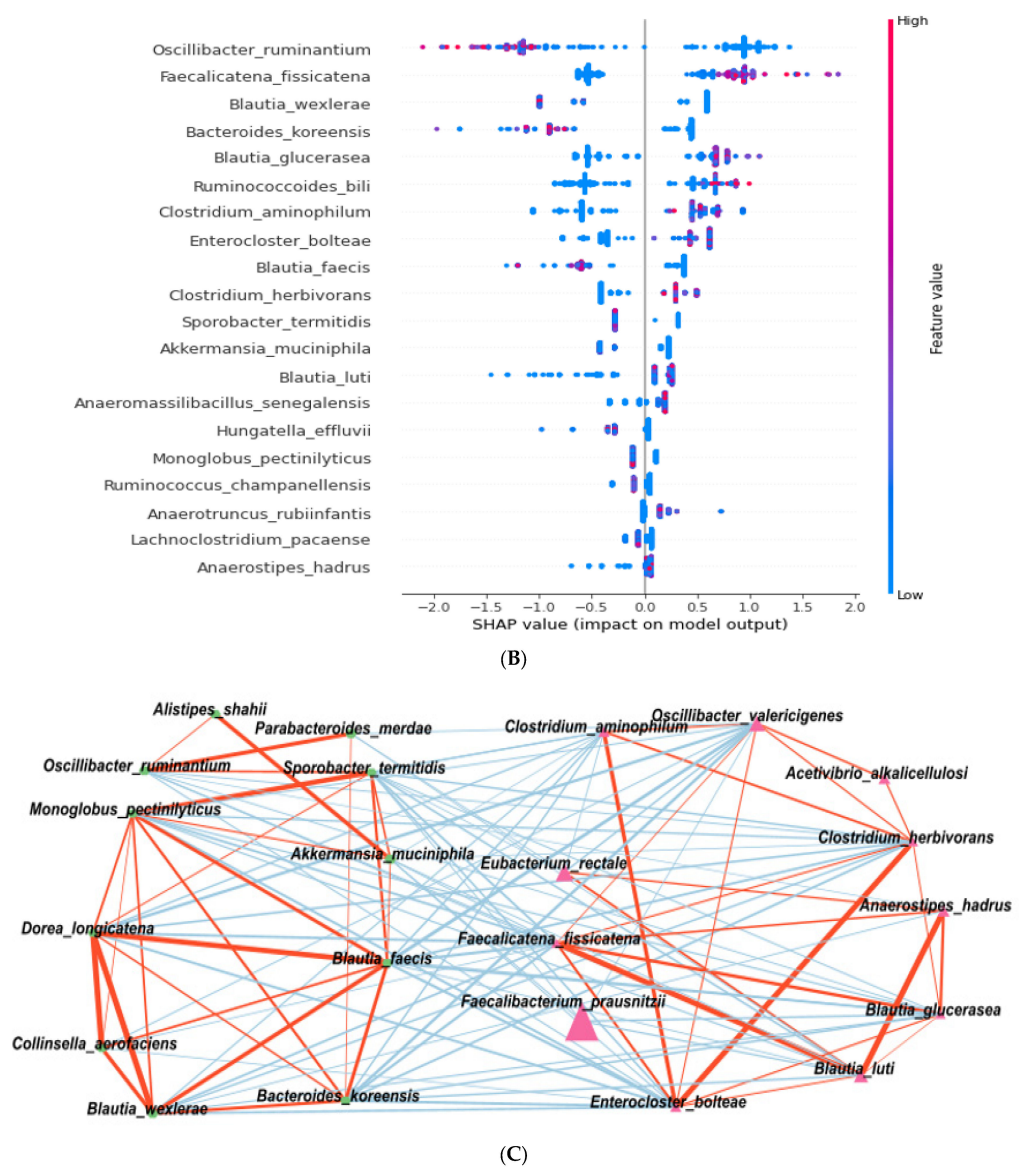
Fecal bacteria composition in Enterotype Lachnospiraceae (ET-L). The distinct differences in the abundance and interactions of specific fecal bacteria between individuals with T2DM and the healthy group within the ET-L enterotype. ET-L participants in the T2DM group had a higher abundance of genera *Bacteroides*, *Phocaeicola*, *Allistipes*, *Blautia*, *Oscillibacter*, and *Ruminococcus* compared to the healthy group. Unlike in ET-B, bacteria in both the T2DM and healthy groups within ET-L exhibited a more stable and positive interaction within each group, with a negative interaction between the T2DM and healthy groups. (**A**). Relative abundance of fecal bacteria compositions at the genus level between type 2 diabetes (T2DM) and healthy groups. (**B**). Primary bacteria between the type 2 diabetes (T2DM) and healthy groups at the species level by XGboost in each enterotype. (**C**). Network of primary bacteria in the T2DM and healthy groups according to each enterotype. Red and blue lines indicate positive and negative correlations with Pearson correlation coefficient >0.1, respectively (*p* < 0.001). * Significant differences between T2DM and healthy groups at *p* < 0.00001 (Bonferroni corrected *p* value).

Within ET-L, among the families present, the proportion of *Bacteroidaceae* and *Lachnospiraceae* was higher, but *Clostridiaceae* was lower in the T2DM than in the healthy group. The participants in the T2DM group had a higher abundance of genera *Bacteroides*, *Phocaeicola*, *Allistipes*, *Blautia*, and *Oscillibacter* than the healthy group, while that of *Ruminoccocus* was higher in T2DM (*p* < 0.0001; Figure 4A). The α-diversity, calculated with the ACE, Chao1, and Shannon indexes, was higher, and that calculated with the Simpson index was lower in the healthy group than the T2DM group (*p* < 0.0001; Supplementary Figure S3C). This indicated that the fecal bacteria were more diverse in the healthy group than the T2DM group. The bacterial community was clearly separated in the healthy and T2DM groups with a *p* < 0.001 (Supplementary Figure S4C). 

The SHAP model showed that the bacterial communities in the T2DM and healthy groups were distinct, while the AUROC was 1.0 in XGBoost, 1.0 in a random forest, and 0.997 in linear regression, indicating that the models for the bacterial composition of the T2DM and healthy groups were perfect using XGBoost, random forest, and linear regression (Table 1). 

The fecal bacteria in the T2DM group showed a higher abundance of *Enterocloster bolteae, Oscillibacter valericigenes, Blautia glucerasea*, and *Faecalicatena fissicatena. Eubacterium rectale, Faecalibacterium prausnitizii, Blautia luti,* and *Streptococcus salivarius* than those in the healthy group (*p* < 0.001). The fecal bacteria of the healthy group showed a higher abundance of *Oscillibacter ruminantium, Bacteroides koreensis, Blautia wexlerae, Akkermentia muciniphila, Bacteroides uniformis,* and *Phascolarctobacterium faecium* than the T2DM group (*p* < 0.001; Figure 4B). 

Similar to ET-B, in ET-L, the bacteria in the healthy group were positively associated with each other, but negatively interacted with those in the T2DM group (*p* < 0.001; Figure 4C). However, unlike ET-B, bacteria in the T2DM and healthy groups within ET-L showed a more stable and positive interaction within each group and a negative interaction between the T2DM and healthy groups. 

### 2.5. Differences in the Bacterial Composition of the T2DM and Healthy Groups in ET-P

**Figure 5:**
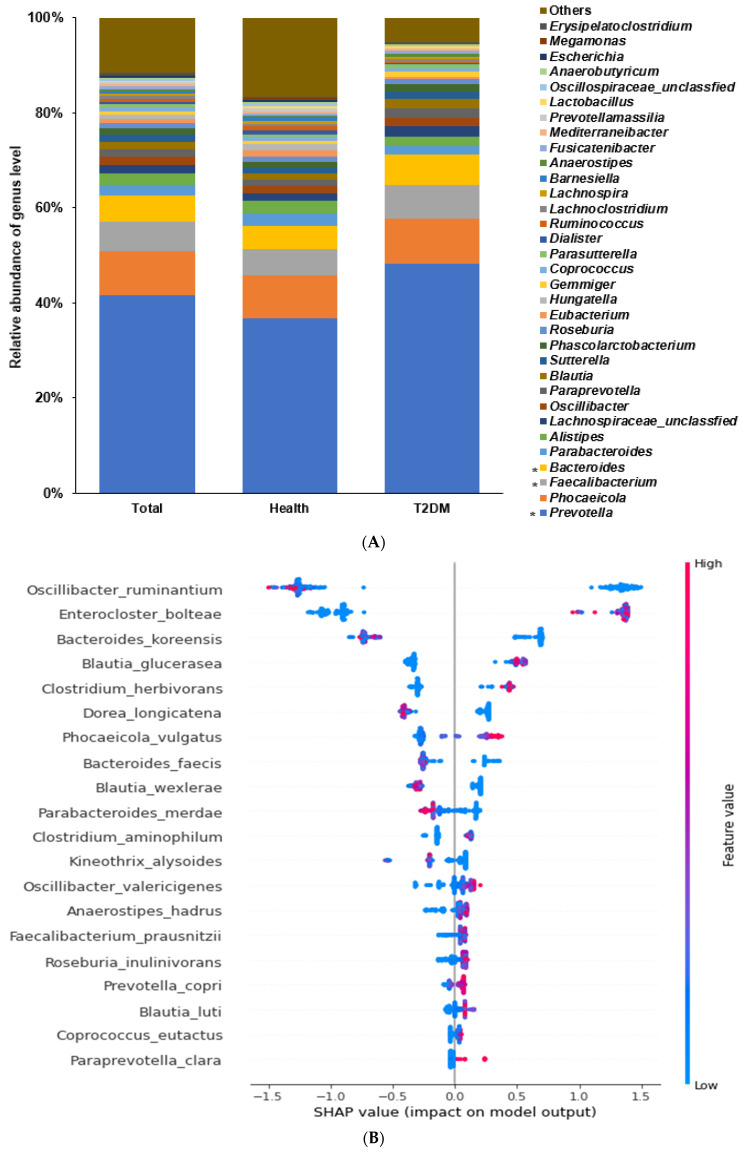

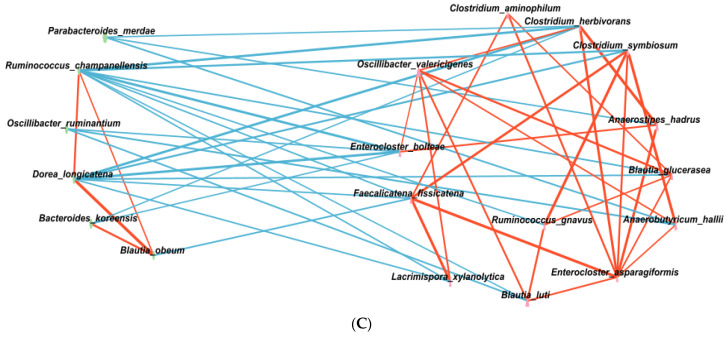
Fecal bacteria composition in Enterotype Prevotellaceae (ET-P). The distinct differences in the proportion and interactions of specific bacteria between individuals with T2DM and the healthy group within the ET-P enterotype. ET-P US adults with T2DM had a higher proportion of genus *Prevotella*, *Facalibacterium*, and *Bacteroides* than healthy adults. In ET-P, similar to ET-B, the bacteria within the T2DM group demonstrated a more stable interaction than those in the healthy group. However, the interaction within the T2DM group in ET-P was less stable than in ET-B. (**A**). Relative abundance of fecal bacteria compositions at the genus level between type 2 diabetes (T2DM) and healthy groups. (**B**). Primary bacteria between the type 2 diabetes (T2DM) and healthy groups at the species level by XGboost in each enterotype. (**C**). Network of primary bacteria in the T2DM and healthy groups according to each enterotype. Red and blue lines indicate positive and negative correlations with Pearson correlation coefficient > 0.1, respectively (*p* < 0.001). * Significant differences between T2DM and healthy groups at *p* < 0.00001 (Bonferroni corrected *p* value).

Within ET-P, those in the T2DM group had a higher proportion of the family Prevotellaceae and Lachnospiraceae and genus *Prevotella*, *Facalibacterium*, and *Bacteroides* compared to those in the healthy group (*p* < 0.0001; Figure 5A). The Chao1 and Shannon indexes were higher, but the Simpson index was lower in the healthy group than in the T2DM group (*p* < 0.0001; Supplementary Figure S3D). These results indicate that the fecal bacterial compositions were more diverse in the healthy group than in the T2DM group. The bacterial community was clearly demarcated into the healthy and T2DM groups with a *p* < 0.001 (Supplementary Figure S4D). The β-diversity determined by the Bray-Curtis dissimilarity matrix was significantly different between the healthy and T2DM groups (*p* < 0.001).

The SHAP model showed that the bacterial communities in the T2DM and healthy groups were distinct and separate: the AUROC was 1.0 in XGBoost, 1.0 in a random forest, and 0.951 in linear regression, indicating that the models for bacterial composition between the T2DM and healthy groups fitted well (Table 1). 

The T2DM group had a higher proportion of Enterocloster bolteae, Blautia glucerases, Clostridium herbivorans, Phocaeicola vulgatus, Oscillibacter valericigenes, Fecalibacterium prausnitizii, Prevotella copri, and Blautia luti than those in the healthy group (*p* < 0.001). The healthy group had a higher proportion of Oscillibacter ruminantium, Bacteroides koreensis, Dorea longicatena, Blautia wexlerae, and Parabacteroides merdae than the T2DM group (*p* < 0.001; Figure 5B). 

The bacteria in the healthy group were positively associated with each other but interacted negatively with those in the T2DM group (*p* < 0.001; Figure 5C). Similar to ET-B, ET-P bacteria within the T2DM group showed a more stable interaction than those in the healthy group. However, the interaction between the bacteria within the T2DM group was less stable in the ET-P than in the ET-B. 

### 2.6. Metagenome Function of Fecal Bacteria

**Figure 6:**
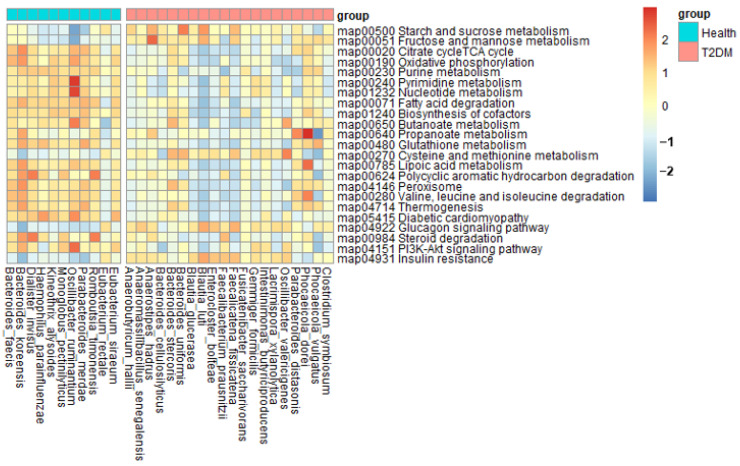
Metagenome functions of the primary bacteria in enterotype Bacteroidaceae (ET-B). Fecal bacteria in T2DM of US adults with ET-B contributed to elevated insulin resistance by decreasing insulin signaling pathways and increasing glucagon signaling. The metabolic differences observed provide insights into the dysregulated insulin resistance associated with T2DM.

Fecal bacteria associated with T2DM was similar in all enterotypes, and metagenome function was analyzed with fecal bacteria higher or lower in the T2DM group than in the healthy group (*p* < 0.0001; Figure 6). In the T2DM group, glucose metabolism, including starch, sucrose, fructose, and mannose metabolism, and cysteine and methionine metabolism was elevated compared to the healthy group. In addition, insulin resistance-related metabolism and glucagon signaling pathway increased in T2DM (*p* < 0.0001; Figure 6). However, fatty acid-related energy metabolism, antioxidant-related metabolism, purine metabolism, and branched-amino acid degradation were elevated in the healthy group rather than in the T2DM group. Insulin signaling pathway and steroid degradation were also elevated in the healthy group compared to the T2DM group (*p* < 0.0001; Figure 6). Furthermore, propanoate and butanoate metabolisms were lower in the T2DM group than in the healthy group (Figure 6). Gut bacteria in T2DM elevated insulin resistance by decreasing insulin signaling and increasing glucagon signaling.

## 3. Discussion

Gut microbiota related to T2DM have been studied, but the results are inconsistent. In this study, we investigated the association between fecal bacterial composition, enterotypes, and T2DM in US adults, focusing on enterotypes. Our objective was to provide valuable insights into the role of gut microbiota in T2DM pathogenesis, specifically within different enterotypes, and to shed light on the link between gut microbiota and T2DM in the US population. Using a large dataset pooling fecal bacterial files of 1039 individuals with T2DM and 872 healthy adults from HMP data, we employed machine learning and network analysis to identify key bacteria and their interactions influencing T2DM incidence. Additionally, we conducted a metagenomic analysis to explore the functional implications of the microbial composition. Our findings revealed distinct enterotypes (Bacteroidaceae, Lachnospiraceae, and Prevotellaceae) associated with T2DM incidence, along with lower α-diversity in T2DM within specific enterotypes, indicating microbial diversity differences. The XGBoost model demonstrated high accuracy and sensitivity in predicting T2DM based on fecal bacterial composition. Furthermore, we observed specific bacterial taxa in the T2DM group compared to the healthy group, and metagenomic analysis unveiled associations between bacterial abundance and metabolic pathways relevant to T2DM. These novel findings enhance our understanding of the intricate interplay between gut microbiota and T2DM in the US population.

Notably, Asians exhibit lower insulin-secreting capacity than non-Asians, contributing to the etiological differences in T2DM development between these populations [3]. The different etiology may be closely linked to distinct gut microbiota communities in Asians and non-Asians. The present study explored the differences in gut microbiota composition between individuals with T2DM and healthy individuals in the USA, predominantly Caucasians. It showed that the enriched bacteria in the T2DM patients in the USA were *Bacteroides*, *Blautia*, and *Germmiger.* However, in Asians, the T2DM-enriched genera are *Enterobacter*, *Coprococcus*, *Negativibacillus*, *Rothia*, *Desulfovibrio*, *Megasphaera*, *Eubacterium Prevotella*, *Clostridium sensu stricto 1*, *Olsenella*, *Lactobacillus*, and *Neisseria*. *Coprobacter*, *Butyrivibrio*, *Paraprevotella*, *Tyzzerella 3*, and *Barnesella* belong to T2DM-depleted genera [11]. Furthermore, Asians with T2DM displayed a higher proportion of *Escherichia fergusonii* and lower *Faecalibacterium prausnitzii* compared to the healthy group within both ET-L and ET-P enterotypes [11]. These differences in gut bacteria between the healthy and T2DM groups in the Asian population highlight the potential association of gut dysbiosis with intestinal permeability and the enteric vagus nervous system [11]. Activation of the enteric vagus nervous system in the intestines can generate aberrant signals to the hypothalamus, leading to a distorted efferent message that induces insulin resistance [17]. Unlike the findings in the Asian population containing ET-L and ET-P, the fecal bacteria in the US population were clustered into ET-B, ET-L, and ET-P, and T2DM-related fecal bacteria did not appear to be associated with intestinal permeability and the enteric vagus nervous system. In the metagenomic analysis, unlike healthy adults, the high fecal bacteria in T2DM patients were implicated in reduced energy utilization, butanoate and propanoate metabolism, and insulin signaling pathways. These findings suggest that the gut bacteria associated with T2DM in the US population primarily relate to energy metabolism and insulin resistance.

Several studies have reported a significant difference in the gut microbiota profiles across ethnicities in the US population [18]. The α-diversity plays a critical role in disease prevalence, and the α-diversity (Choa1 and Shannon indexes) is reported to be lower in the order of ET-L, ET-P, and ET-B in healthy persons [19]. It suggests that ET-B may be more susceptible to metabolic diseases, including T2DM. However, the decrement in α-diversity in T2DM remains controversial [11,20]. A systematic review and meta-analysis of stool microbial profiles, including seven studies, involving 600 T2DM patients and 543 controls from China, Pakistan, Mexico, Columbia, and Nigeria, showed significant β-diversity but not α-diversity between the T2DM and control groups as shown in a random effect model [20]. In the other Asian study separated into ET-P and ET-L clusters, α-diversity is lower in the T2DM group than the healthy group in ET-L, but not ET-P [11]. The present study demonstrated that α-diversity was lower in the T2DM group than the healthy group in the total participants. A similar trend was seen in ET-L and ET-P, but not ET-B. These results suggest that the population with no difference between the healthy and T2DM groups may be susceptible to T2DM. Therefore, Asians with ET-P and US adults with ET-B may be at a high risk of T2DM. ET-B may benefit by switching to a different enterotype. 

Enterotypes are linked to not only the host’s genetic factors, but also diets [11,14,21,22]. Asians have consumed a low-fat diet with grains and vegetables and have ET-L or ET-P, but not ET-B. *Prevotella* and *Bacteroides* belong to Bacteroidetes in the phylum level, and a high-fat diet partly changes ET-P to ET-B [23]. Although enterotypes are challenging to alter [24,25], dietary patterns are the primary drivers of enterotypes, regardless of other factors. Overall, ET-B is mainly related to a high-fat and protein diet from animal foods, ET-P is linked to insufficient energy intake, simple sugars, fruits, and vegetables, and ET-L is involved with a mixed diet, possibly a balanced diet [19]. Bile acid acts as a molecular cross-talk between gut microbiota and the host, and the bile acid pool in the colon modulates gut microbial metabolism and diversity [26]. A high-fat diet, especially with gallbladder removal, has been shown to elevate *Bacteroides* and decrease α-diversity in mice [21]. In contrast, irritable bowel disease is linked to high bile acid content and a lower proportion of *Bacteroides* [27]. It suggests that elevated bile acid may promote a gut condition to increase *Bacteroides*. In recent years, the sudden increase of T2DM in Asians may be related to increasing Bacteroides, although enterotypes cannot be easily altered. US adults have a high-fat diet with high meats and low vegetables and more ET-B than Asians. Since US adults with ET-B showed no difference in α-diversity between the T2DM and healthy groups, they may be susceptible to T2DM. Therefore, US adults may need to make dietary modifications to decrease *Bacteroides*. 

T2DM is involved in increased insulin resistance and insufficient insulin secretion, which are linked to gut microbiota [ref]. In the Rotterdam study, patients with T2DM and high insulin resistance (high HOMA-IR) exhibited a lower Shannon index and richness than those without T2DM. A higher abundance of Christensenellaceae, *Marvinbryantia*, and Ruminococcaceae was inversely associated with insulin resistance [28]. A higher abundance of Clostridiaceae, *Intestinibacter*, and Peptostreptococcaceae was inversely associated with the incidence of T2DM. These bacteria were involved in butyrate production [28]. The present study showed that *Enterocloster bolteae*, *Facalicatena fissicatena*, *Clostridium symbiosum*, and *Facalibacterium prausnitizii* were present in higher abundance, and *Bacteroides koreensis*, *Oscillibacter ruminantium*, *Bacteroides uniformis*, and *Blautia wexlerae* were present in lower numbers in the T2DM group compared to the healthy group in the USA, regardless of enterotypes. *Akkermentia muciniphila* was higher in the healthy group than the T2DM group only in ET-L. In a metagenomic analysis of fecal bacteria, the predominant bacteria in T2DM from the USA were related to reduced energy utilization, decreased butanoate and propanoate metabolism, and disturbed insulin signaling pathways compared to healthy adults, somewhat different from those associated with Asian T2DM. 

Each bacterium may or may not be related to T2DM in a cause-and-effect relationship. It is not individual bacteria, but their group influencing glucose metabolism and progression to T2DM. *Facalibacterium prausnitizii* and *Bacteroides uniformis* act as probiotics to promote health benefits [29,30]. However, the present study demonstrated that these bacteria were present in higher numbers in participants with T2DM than in healthy adults. For example, *Facalibacterium prausnitizii* and *Bacteroides uniformis* could be involved in the development and progression of T2DM since the results of the present study came from case-control studies and not randomized clinical trials. Gut microbial networking has been studied mainly in the context of infections since co-existent bacteria significantly prevent infections and are not detrimental to the host [31]. However, few studies have been conducted to investigate gut microbial interactions to prevent metabolic diseases, and these studies have not been conducted according to enterotypes [32]. A stable bacterial network can prevent gut dysbiosis, which in turn can prevent disease progression. Probiotics and prebiotics should regulate the gut bacterial network to prevent disease development and progression. Gut bacteria are better at promoting butyrate-producing bacteria, such as *Akkermansia muciniphila*, to prevent the development of T2DM [8,9]. 

This study has some merits. First, the study included a large sample size by pooling all studies conducted with US adults (1039 T2DM and 872 healthy adults). Second, the gut microbiota in US adults showed a distinct gut microbiota community different from Asians. Dysfunction of the parasympathetic nervous system can develop and exacerbate T2DM, especially in Asians, contributing to gut dysbiosis [33]. The present study provides evidence that T2DM patients in the USA were not related to suppressing the parasympathetic nervous system, but linked to insulin signaling pathways and energy metabolism, reiterating the etiological differences in T2DM between Asians and Caucasians [34]. This contributes to understanding T2DM etiology and highlights potential implications for personalized interventions or treatments. However, the present study also has some limitations. First, the data were collected in case-control studies, and the results could not be applied to evaluate cause and effect. Second, the fecal FASTA/Q data from the adults in the USA were collected from the GMrepo database. However, the demographic characteristics of the participants, such as age, gender, ethnicity, food intake, and lifestyle, were not provided and hence could not be used for adjustments in the statistical analysis. Information on the drugs used to treat T2DM and antibiotic intake was also not provided. Third, the direct effect of insulin secretion, insulin resistance, and the parasympathetic nervous system on gut microbiota could not be evaluated due to the non-availability of biochemical data. 

In summary, the fecal bacterial composition of US adults with T2DM was clearly separated from those of healthy participants. Regardless of enterotypes, *Enterocloster bolteae*, *Facalicatena fissicatena*, *Clostridium symbiosum*, and *Facalibacterium prausnitizii* were present in higher abundance, and *Bacteroides koreensis*, *Oscillibacter ruminantium*, *Bacteroides uniformis*, and *Blautia wexlerae* were present in lower numbers in the T2DM group compared to the healthy group in the XGBoost model. The gut microbiota in adults with T2DM in the USA was somewhat different from those in Asians with T2DM, which may be linked to different β-cell functions and mass in Asians and non-Asians. The interaction between the fecal bacteria was also different in different enterotypes. ET-L had a more stable gut microbiota population than ET-B, and adults with ET-P and ET-L had a lower T2DM incidence than those with ET-B. In the metagenomic analysis, gut bacteria composition in T2DM patients was inversely associated with energy utilization, butanoate and propanoate metabolism, and insulin signaling pathways compared to healthy adults. In conclusion, the gut bacteria related to T2DM mainly influence the energy metabolism and insulin signaling pathways in the US population, Caucasians, and the regulation of their network is linked to T2DM risk in different enterotypes. Therefore, the present study provides valuable insights into the link between gut microbiota and T2DM in US adults. This contributes to understanding T2DM etiology and highlights potential implications for personalized interventions or treatments.

## 4. Methods

### 4.1. Collection and Pooling of Fecal Bacteria FASTA/Q Files for Healthy and T2DM Adults 

Figure 1 outlines the overall analysis process of fecal bacteria in T2DM by pooling the FASTA/Q files of the fecal bacteria. The files were collected from the projects that studied the fecal bacterial composition of T2DM patients downloading the NCBI SRA database (https://www.ncbi.nlm.nih.gov/sra (accessed on 6 March 2022)) and the Human Microbiome Project (https://portal.hmpdacc.org/ (accessed on 10 March 2022)), organized and funded by the National Institutes of Health (NIH) in the United States. The National Human Genome Research Institute (NHGRI), the National Institute of Allergy and Infectious Diseases (NIAID), the National Institute of Dental and Craniofacial Research (NIDCR), and the National Institute of Diabetes and Digestive and Kidney Diseases (NIDDK), among others in the USA, had a collaboration to collect and analyze samples from various sites. In the present study, the selection criteria for the samples were as follows: (1) Host: *Homo sapiens*, (2) Sample type: human feces, (3) Target participants: Caucasians having T2DM aged over 30 years, (4) Control participants: US healthy adults (without T2DM), (5) Assay: amplicon sequencing (Miseq), (6) Target sequencing: 16S rRNA. Participants volunteered to provide fecal samples in each project which the corresponding Institutional Review Board approved of the respective institute. They signed informed consent forms. The fecal FASTA/Q files were collected from the studies conducted mainly in the USA, and the participants were 872 healthy adults and 1039 adults with T2DM (Table 1). The participants provided their ages and gender. 

### 4.2. Fecal Bacterial Composition and Community Analysis 

The FASTA/Q files from the Caucasian fecal samples that satisfied the inclusion criteria were downloaded and extracted using the National Center for Biotechnology Information (NCBI) Sequence Read Archive (SRA) toolkits (https://trace.ncbi.nlm.nih.gov/Traces/sra/sra.cgi?view=software (accessed on 20 March 2022)). The collected fecal FASTA/Q files were processed using qiime2 (https://qiime2.org/ (accessed on 20 April 2022)). In brief, the sequences of the collected files were merged and aligned with the SILVA v 1381 database. Bacterial sequences were collected by removing non-target sequences, including mitochondria, archaea, fungi, and unknown sequences. The fecal bacterial sequences were preclustered, and chimeras were eliminated. The remaining sequences were clustered with 97% similarity, and the taxonomy of the operational taxonomic units (OTUs) was annotated according to the NCBI Basic Local Alignment Search Tool (BLAST) (https://blast.ncbi.nlm.nih.gov/Blast.cgi (accessed on 3 May 2022)). The biome file containing the taxonomy and counts was used for further analysis. 

### 4.3. Enterotypes 

The fecal bacteria were clustered by principal component analysis (PCA) using the taxonomy and counts of the biome file. The number of clusters was designated by satisfying eigenvalues >1.5 using the R PCoA package (https://cran.r-project.org/web/packages/aPCoA/index.html, accessed on 18 May 2022) [19]. 

### 4.4. α-Diversity, β-Diversity, and Linear Discriminant Analysis (LDA) Scores of Fecal Bacteria 

The types and diversity of fecal bacteria bidirectionally influence host metabolism and health status. Alpha-diversity represents the diversity of the bacteria in a host’s gut assessed by the Chao1, Shannon, Simpson, and Ace indexes. Beta-diversity is the ratio between regional and local species diversity that demonstrates group separation [35]. The α- and β-diversities of the bacteria were calculated using the Mothur software package, and the results were visualized with the R program. The statistical difference of β-diversity between the healthy and T2DM groups was checked with permutational multivariate analysis of variance (PERMANOVA). The LDA scores represent the effect size of each abundant species, and they were analyzed with the lefse command in the Mothur program. 

### 4.5. Extreme Gradient Boosting (XGBoost) Classifier Training and SHapley Additive exPlanations (SHAP) Interpreter

According to the enterotype, the specific predominant fecal bacteria in the T2DM group were analyzed with a machine learning approach, including XGBoost, random forest, and linear regression, and compared with the healthy group. The fecal data were divided randomly into 80% for the training and 20% for the testing sets. The hyperparameter settings were a random grid search with 1000 iterations of the XGBoost algorithm using the Scikit package (https://xgboost.readthedocs.io/en/stable/install.html, accessed on 25 May 2022) [36]. The models for explaining the healthy and T2DM groups were generated. The receiver operating characteristic (ROC) area for the models was determined using the training and test sets of the fecal bacteria. The 10-fold cross-validation was calculated using the “cross_val_score” function in the test data set. The original training data were clustered into 10 subsets for the calculation: eight sets were used as training data and two sets as test data, and they were iterated ten times [37]. The accuracy, specificity, and sensitivity were calculated from the data sets. The value of the 10-fold cross-validation indicated the accuracy of the selected model [38]. 

The SHAP analysis was conducted with the SHAP (0.39.0) package from https://shap.readthedocs.io/en/latest/index.html (accessed on 8 June 2022) to separate the bacteria positively or negatively involved in the T2DM group using the output of the XGBoost model [38,39]. The correlation of the bacterial species was carried out using the Pearson correlation analysis. Network analysis was conducted using the correlation results to investigate the gut microbes using the Cytoscape program downloaded from the website (https://cytoscape.org/ (accessed on 20 June 2022)). 

### 4.6. Metagenome Function of Fecal Bacteria by Phylogenetic Investigation of Communities by Reconstruction of Unobserved States (Picrust2)

The metagenome functions of the fecal bacteria were estimated from the genes they contained using the FASTA/Q files. These functions were determined with the Picrust2 software for predicting functional abundances based only on marker gene sequences [40]. The metabolic functions of the genes in the fecal bacteria were estimated based on the Kyoto Encyclopedia of Genes and Genomes (KEGG) Orthologues (KO) and mapped using the KEGG mapper (https://www.genome.jp/kegg/tool/map_pathway1.html (accessed on 3 July 2022)) [40]. 

### 4.7. Statistical Analysis

The statistical analysis used SAS version 7 (SAS Institute; Cary, NC, USA) and the R package version 4.2.0. The data were expressed as the mean ± standard deviation (SD). To assess the mean statistical differences between the T2DM and healthy groups based on enterotypes, a two-sample *t*-test was conducted with a Bonferroni correction applied to the *p* values. Additionally, a one-way ANOVA with a Bonferroni correction was performed to examine the mean differences among the three enterotypes. Visualization of the data was conducted using R-studio and the ggplot2 package. 

## Figures and Tables

**Table 1:** Accuracy, sensitivity, specificity, and precision of the prediction models according to enterotypes.

Total Participants	AUROC	Accuracy	Sensitivity	Specificity	Precision	F1
XGBoost	1.0 ± 4.1 × 10^−6^	9.9 ± 1.7 × 10^−4^	0.99 ± 2.5 × 10^−4^	0.99 ± 2.0 × 10^−4^	1.0 ± 1.6 × 10^−4^	0.99 ± 1.52 × 10^−4^
Random forest	1.0 ± 0.001	1.0. ± 8.2 × 10^−5^	1.0 ± 0.0	0.99 ± 1.9 × 10^−4^	1.0 ± 1.5 × 10^−4^	1.0 ± 7.36 × 10^−5^
Linear regress	0.99 ± 2.0 × 10^−4^	0.97 ± 2.0 × 10^−4^	0.97 ± 3.4 × 10^−4^	0.96 ± 4.6 × 10^−4^	0.97 ± 4.6 × 10^−4^	0.97 ± 2.55 × 10^−4^
ET-B	AUROC	Accuracy	Sensitivity	Specificity	Precision	F1
XGBoost	1.0 ± 1.1 × 10^−5^	0.97 ± 3.9 × 10^−4^	0.96 ± 5.3 × 10^−4^	0.97 ± 1.3 × 10^−4^	0.97 ± 0.0005	0.97 ± 0.0004
Random forest	1.0 ± 3.0 × 10^−5^	0.97 ± 3.9 × 10^−4^	0.98 ± 3.9 × 10^−4^	0.97 ± 6.4 × 10^−4^	0.97 ± 0.0005	0.97 ± 0.0003
Linear regress	0.98 ± 2.8 × 10^−4^	0.95 ± 5.2 × 10^−4^	0.97 ± 5.4 × 10^−4^	0.93 ± 7.7 × 10^−4^	0.94 ± 0.0007	0.96 ± 0.0005
ET-L	AUROC	Accuracy	Sensitivity	Specificity	Precision	F1
XGBoost	1.0 ± 0.0	0.99 ± 3.4 × 10^−6^	0.99 ± 3.7 × 10^−4^	1.0 ± 0.0	1.0 ± 0.0	0.99 ± 1.9 × 10^−4^
Random forest	1.0 ± 0.0	0.99 ± 1.9 × 10^−4^	0.99 ± 4.3 × 10^−4^	1.0 ± 0.0	1.0 ± 0.0	0.99 ± 2.3 × 10^−4^
Linear regress	1.0 ± 0.0001	0.98 ± 3.9 × 10^−4^	0.99 ± 4.4 × 10^−4^	0.97 ± 6.8 × 10^−4^	0.98 ± 5.2 × 10^−4^	0.98 ± 3.2 × 10^−4^
ET-P	AUROC	Accuracy	Sensitivity	Specificity	Precision	F1
XGBoost	1.0 ± 0.0	0.98 ± 6.9 × 10^−4^	0.954 ± 0.001	1.0 ± 0.0	1.0 ± 0.0	0.98 ± 8.3 × 10^−5^
Random forest	1.0 ± 0.0	0.96 ± 9.2 × 10^−4^	0.911 ± 0.002	1.0 ± 0.0	1.0 ± 0.0	0.95 ± 0.002
Linear regress	0.95 ± 0.001	0.96 ± 0.001	0.954 ± 0.001	0.957 ± 0.001	0.954 ± 0.001	0.95 ± 0.001

ET-B, enterotype Bacteroidaceae; ET-L, enterotype Lachnospiraceae; and ET-P, enterotype Prevotellaceae. AUROC, the area under the receiver operating characteristic. Precision = TP/(TP + FP). F1 score = 2 × (precision × recall)/(precision + recall) in recall = TP/(TP + FP). TP, true positive value; FP, false positive value; FN, false negative.

## Data Availability

The data to support the findings of this study are available from the corresponding author upon reasonable request.

## Supplementary Materials

The following are available online at https://www.mdpi.com/article/10.3390/ijms24119533/s1.
