# Supplementary material for: Fecal Microbiota Composition, Their Interactions, and Metagenome Function in US Adults with Type 2 Diabetes According to Enterotypes
Source: Int J Mol Sci. 2023 May 31;24(11):9533. doi: 10.3390/ijms24119533 (PMC10253423; doi:10.3390/ijms24119533)
Supplement: Supplementary file 1 [file ijms-24-09533-s001.zip › ijms-2400745-supplementary.pdf]

Supplementary figure

Fig. S1. Enterotype classification by the principal component analysis (PCA)

A. Optimal number of clusters in PCA.

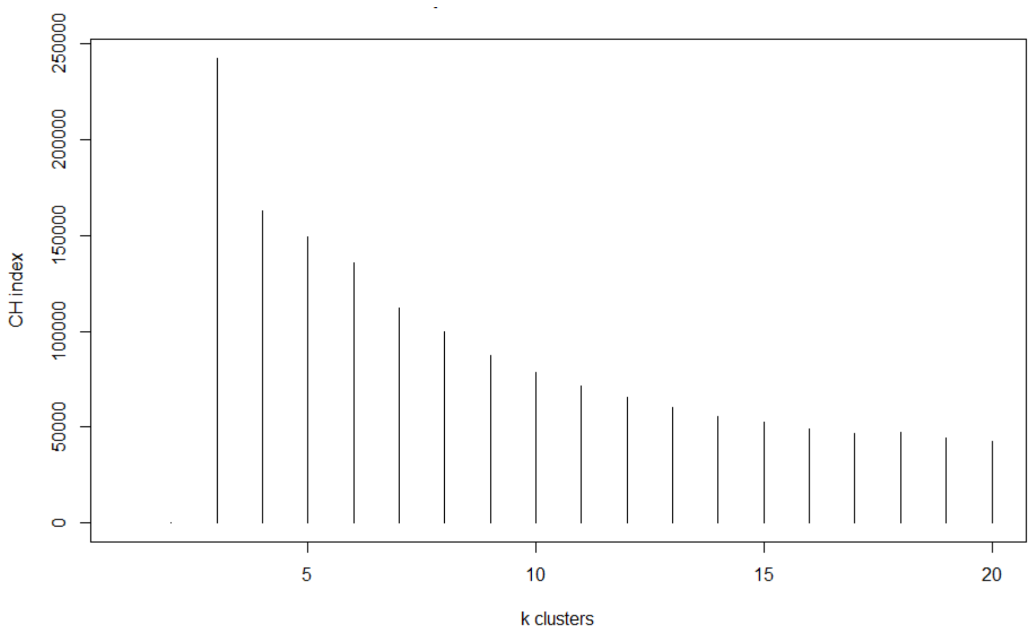

B. Results of principal component analysis

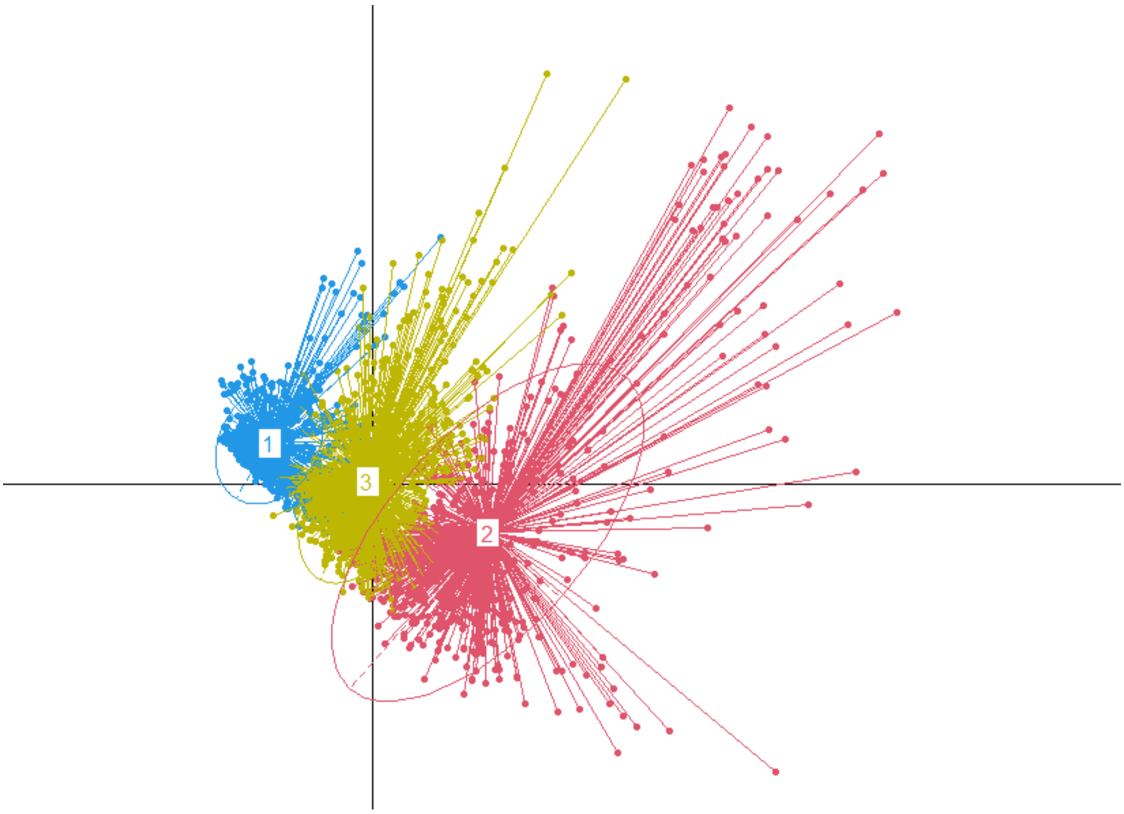

Fig. S2. Fecal bacteria stratification into two enterotypes and bacterial composition in each enterotype

A. Relative abundance of fecal bacteria in each enterotype at the family level

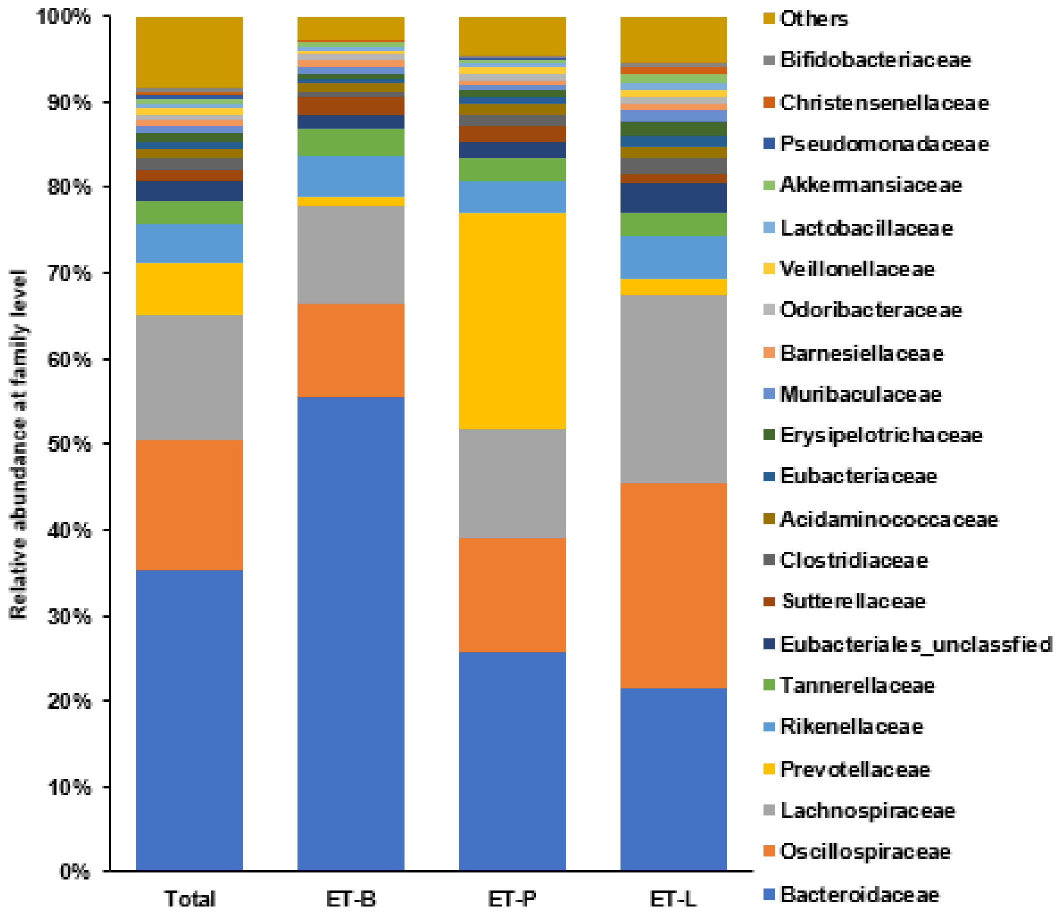

B. Relative abundance of fecal bacteria in each enterotype at the genus level

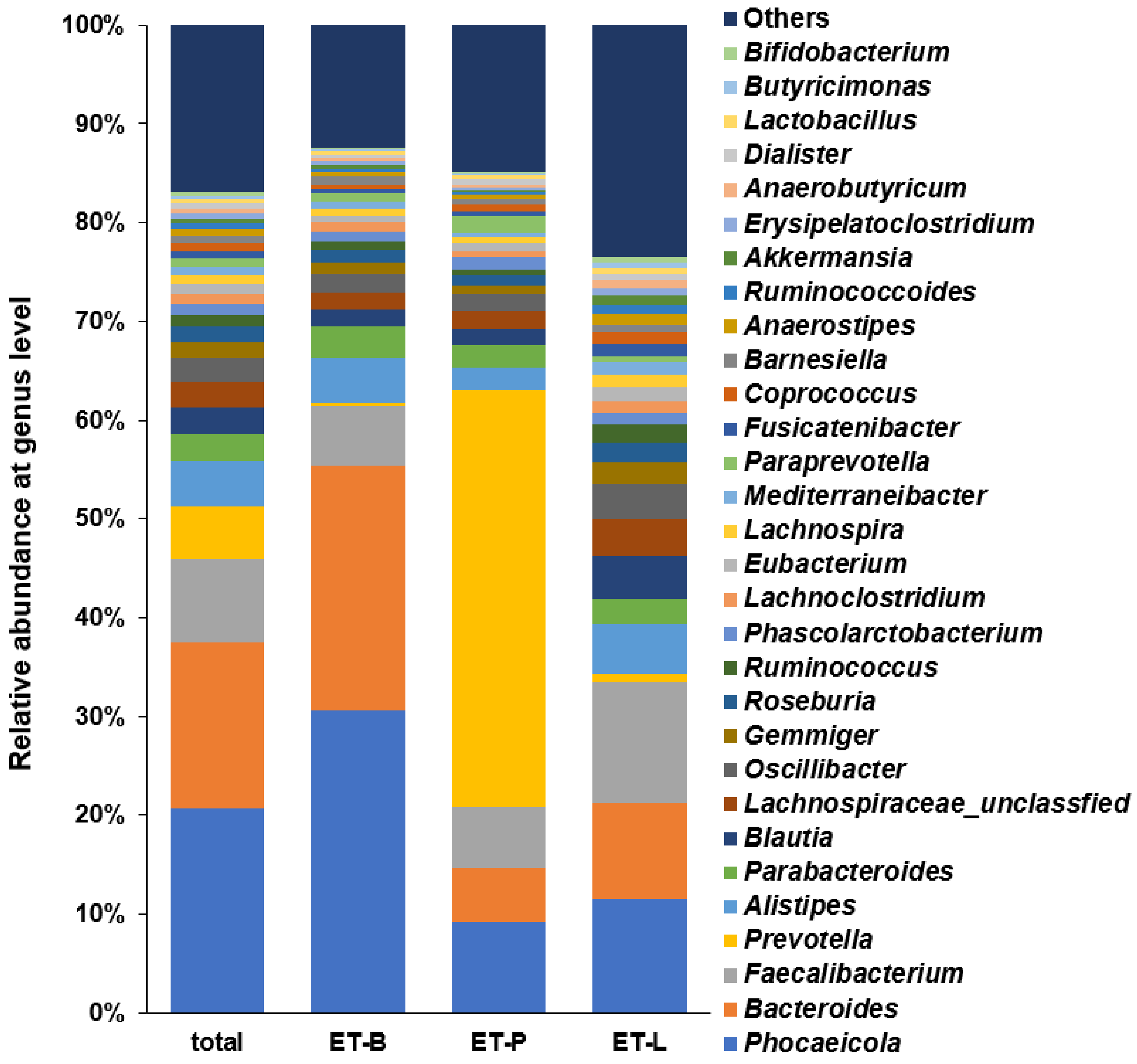

* Significant differences between ET-L and ET-P at P<0.00001 (Bonferroni corrected P value).

ET-L: Enterotype Lachnospiraceae ; ET-P: Enterotype Prevotellaceae

Fig. S3. α-diversity of fecal bacteria

A. α-diversity in total participants

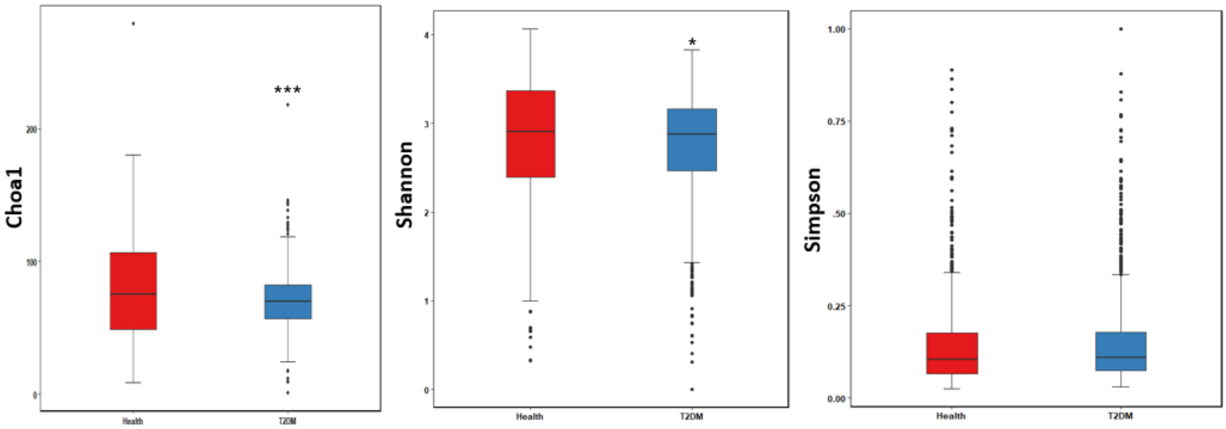

B. α-diversity in ET-B

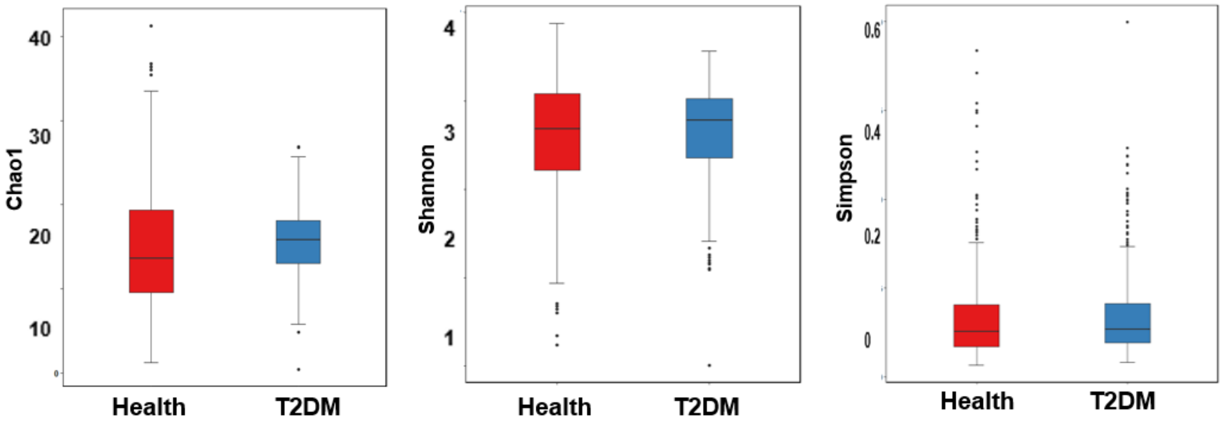

C. α-diversity in ET-L

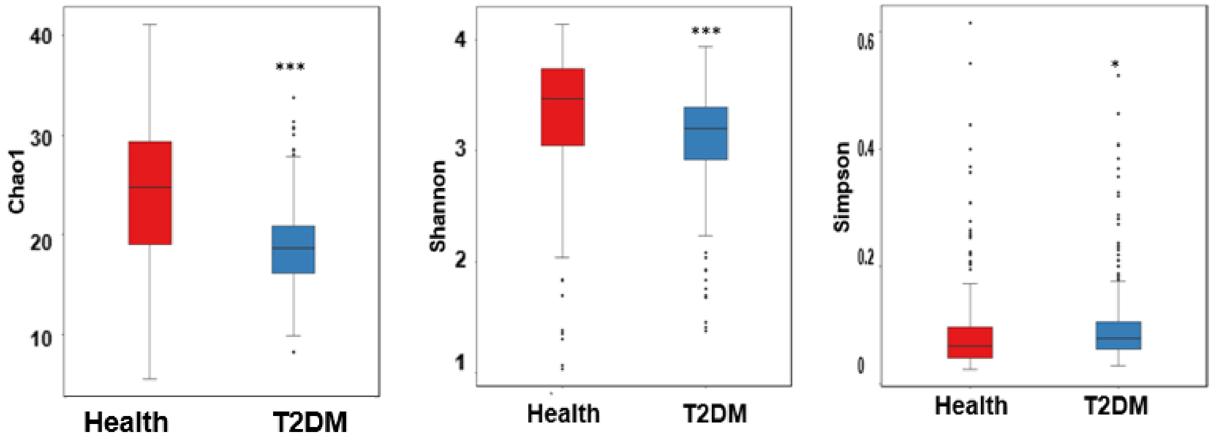

D. α-diversity in ET-P

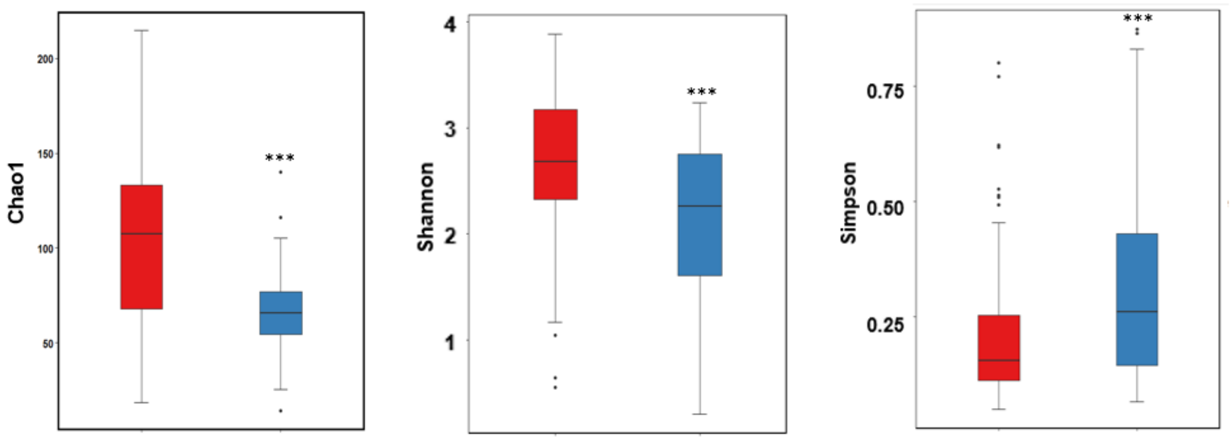
Chao1
200
150
100
50
***
Shannon
4
3
2
1
***
Simpson
0.75
0.50
0.25
***

Fig. S4. β-diversity of fecal bacteria

A. Total participants

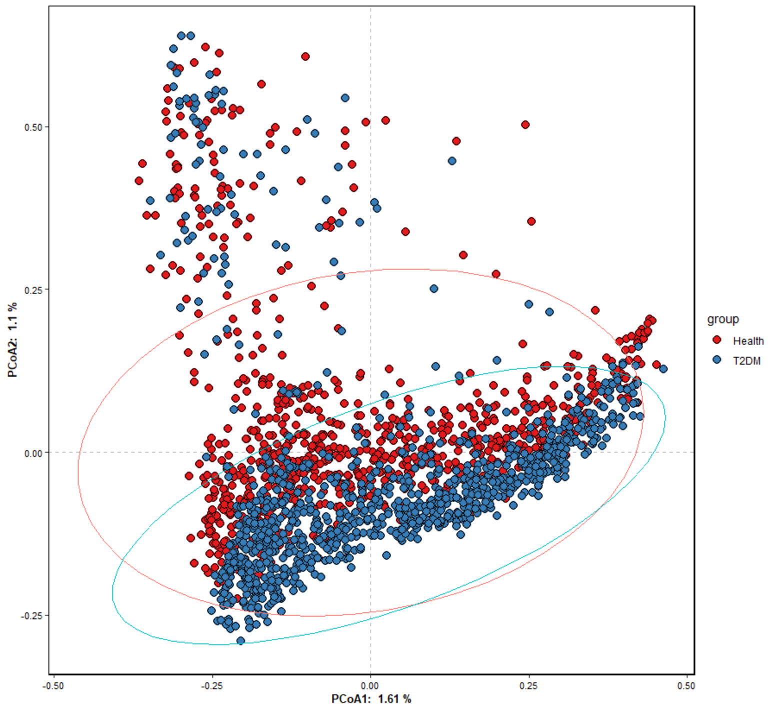

B. ET-B

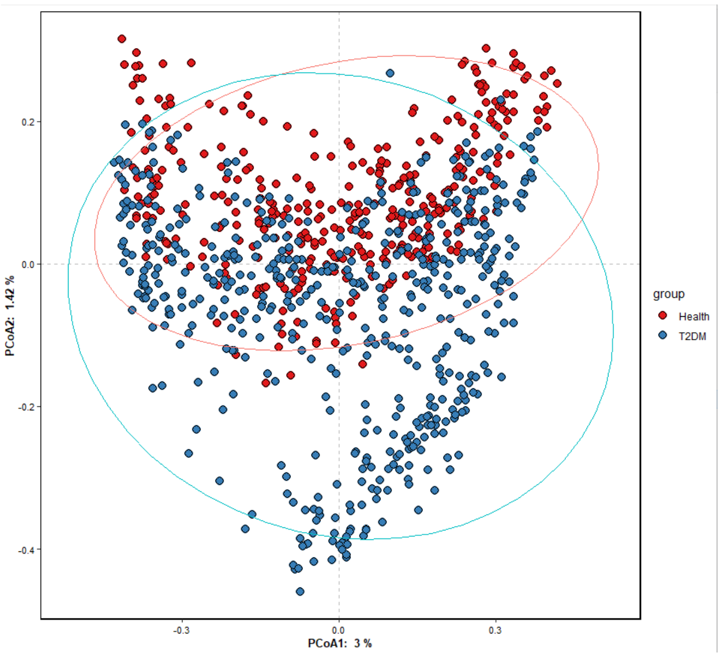

C. ET-L

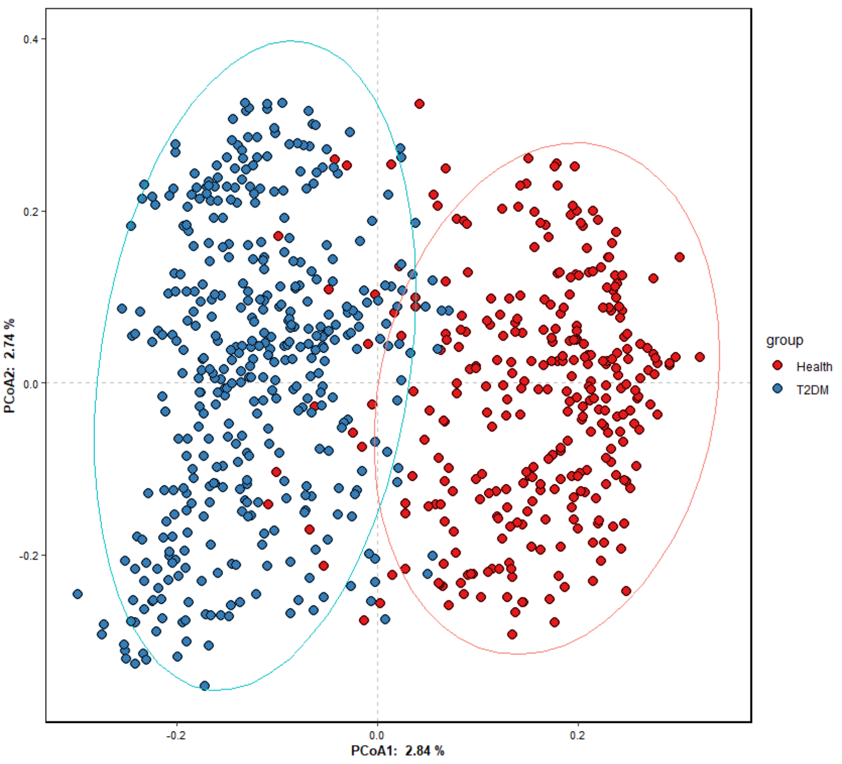
0.4
0.2
0.0
-0.2
-0.2
0.0
0.2
PCoA2: 2.74 %
PCoA1: 2.84 %
group
Health
T2DM

D. ET-P

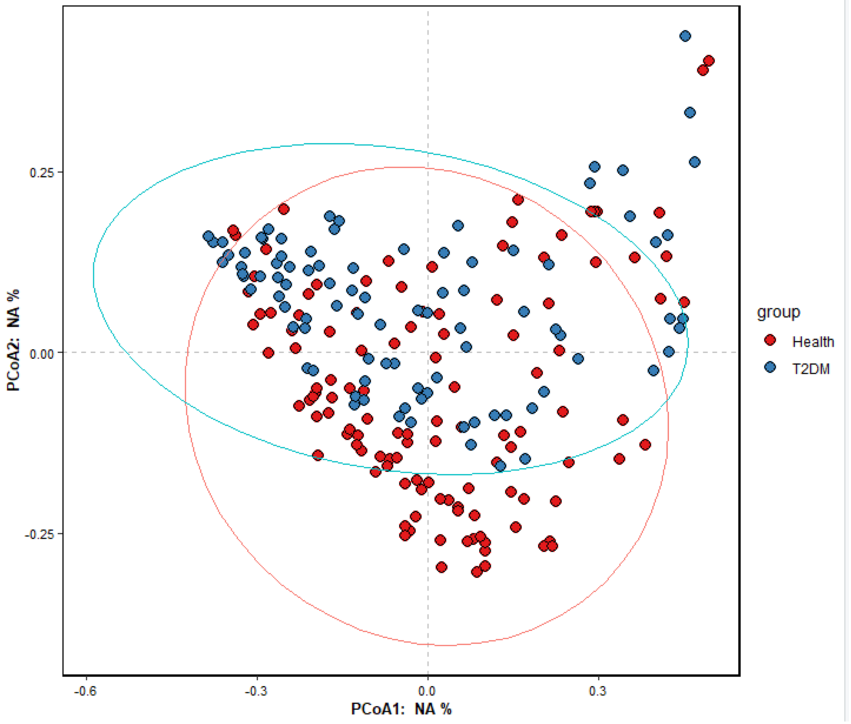
0.25
0.00
-0.25
-0.6
-0.3
0.0
0.3
PCoA2: NA %
PCoA1: NA %
group
Health
T2DM
